# New Agents for Targeting of IL-13RA2 Expressed in Primary Human and Canine Brain Tumors

**DOI:** 10.1371/journal.pone.0077719

**Published:** 2013-10-16

**Authors:** Waldemar Debinski, Peter Dickinson, John H. Rossmeisl, John Robertson, Denise M. Gibo

**Affiliations:** 1 The Brain Tumor Center of Excellence, Thomas K. Hearn Brain Tumor Research Center, Departments of Neurosurgery, Radiation Oncology, Wake Forest School of Medicine, Winston-Salem, North Carolina, United States of America; 2 Department of Surgical and Radiological Sciences, School of Veterinary Medicine, University of California at Davis, Davis, California, United States of America; 3 Department of Small Animal Clinical Sciences and Pathobiology, Virginia-Maryland Regional College of Veterinary Medicine, Virginia Tech, Blacksburg, Virginia, United States of America; The University of Chicago, United States of America

## Abstract

Interleukin 13 receptor alpha 2 (IL-13RA2) is over-expressed in a vast majority of human patients with high-grade astrocytomas like glioblastoma. Spontaneous astrocytomas in dogs resemble human disease and have been proposed as translational model system for investigation of novel therapeutic strategies for brain tumors. We have generated reagents for both detection and therapeutic targeting of IL-13RA2 in human and canine brain tumors. Peptides from three different regions of IL-13RA2 with 100% sequence identity between human and canine receptors were used as immunogens for generation of monoclonal antibodies. Recombinant canine mutant IL-13 (canIL-13.E13K) and canIL-13.E13K based cytotoxin were also produced. The antibodies were examined for their immunoreactivities in western blots, immunohistochemistry, immunofluorescence and cell binding assays using human and canine tumor specimen sections, tissue lysates and established cell lines; the cytotoxin was tested for specific cell killing. Several isolated MAbs were immunoreactive to IL-13RA2 in western blots of cell and tissue lysates from glioblastomas from both human and canine patients. Human and canine astrocytomas and oligodendrogliomas were also positive for IL-13RA2 to various degrees. Interestingly, both human and canine meningiomas also exhibited strong reactivity. Normal human and canine brain samples were virtually negative for IL-13RA2 using the newly generated MAbs. MAb 1E10B9 uniquely worked on tissue specimens and western blots, bound live cells and was internalized in GBM cells over-expressing IL-13RA2. The canIL-13.E13K cytotoxin was very potent and specific in killing canine GBM cell lines. Thus, we have obtained several monoclonal antibodies against IL-13RA2 cross-reacting with human and canine receptors. In addition to GBM, other brain tumors, such as high grade oligodendrogliomas, meningiomas and canine choroid plexus papillomas, appear to express the receptor at high levels and thus may be appropriate candidates for IL-13RA2-targeted imaging/therapies. Canine spontaneous primary brain tumors represent an excellent translational model for human counterparts.

## Introduction

Glioblastoma (GBM) is a high-grade astrocytoma and represents the most common form of primary brain tumor in humans. The successful treatment of patients with GBM is still a major challenge with a median survival rate of 14.5 months after diagnosis [1]. Interleukin 13 receptor alpha 2 (IL-13RA2) is richly over-expressed in GBM [2-4]. This receptor is different from the physiological receptor for IL-13 (IL-4A/IL-13RA1 heterodimer), because it is a monomer and binds only IL-13, and not IL-4, its homologue [5]. IL-13RA2 belongs to a group of cancer/testis like tumor antigens [6] and is one of the downstream gene targets following activation of both wild type EGFR and mutant EGFRvIII [7,8]. Demethylation causes up-regulation of *IL13RA2* suggesting epigenetic mechanisms are also involved in IL-13RA2 receptor regulation [9] in addition to activation of PI3K and ERK pathways [7].

Several molecular therapies targeting IL-13Rα2 have been generated and all have the potential of being applied to management of patients with GBM. Among them are vaccines [10,11],, re-targeted cytotoxic T cells [13], and new rationally designed IL-13 based cytotoxins [14-16]. Additionally, novel IL-13RA2-targeted adenoviral and herpes constructs have been developed and could potentially be used as gene therapy vectors for the treatment of gliomas [12,17,18]. Thus, IL-13RA2 is a truly attractive molecular target, being over-expressed in a majority, but not all patients with GBM [19].

Dogs and humans are the only species in which spontaneously arising primary brain tumors are common. Although the true incidence of canine gliomas is not fully known, the frequency of brain tumors in dogs, based on necropsy data, is similar to humans, i.e. approximately 2% [20]. Prevalence of nervous system tumors in the general population of pet dogs is also similar and has been estimated at 14.5/100,000 animal years [21-24]. Over 70% of primary tumors occur in dogs aged 6 years or more, a period in lifespan comparable to middle age in humans, although they may occur in younger animals as well. Astrocytomas, oligodendrogliomas and invasive meningiomas are most common [25]. High-grade astrocytomas, anaplastic oligodendrogliomas, and mixed anaplastic astrocytic oligodendrogliomas, histomorphologically virtually identical to those seen in humans, have been reported. Unfortunately, patterns of survival for dogs with malignant gliomas are similar to those seen in people, with death relatively soon (weeks to months) after diagnosis, for those animals that are not humanely euthanized immediately, at the time of diagnosis [26].

Tumors of the central nervous system in canine patients are spontaneous, heterogenous, progress over clinically relevant periods of time and are large enough to enable clinically relevant translation of both experimental diagnostic and therapeutic clinical procedures developed recently in human patients [27]. This is particularly important when considering therapeutic approaches using delivery techniques such as convection –enhanced delivery (CED) to large tumor volumes [28-30]. With this translational model in mind the goals of the current study were: a) To further validate canine spontaneous brain tumors as a model system for the investigation of IL-13RA2 targeted therapies, b) To generate MAbs against IL-13RA2 that would be cross-reactive between humans and dogs and be more sensitive than commercially available antibodies, and c) To produce a recombinant cytotoxic agent to target the IL-13RA2 in canine tumors in a species-specific manner.

## Materials and Methods

### Sample collection

All canine tumor tissue was obtained from surgical biopsy/resection specimens, necropsy, or from archival paraffin embedded material from clinical cases presented to the Veterinary Medical Teaching Hospitals, University of California, Davis and Virginia-Maryland Regional College of Veterinary Medicine, and the University of Tennessee College of Veterinary Medicine. All samples were obtained during routine "standard of care" clinical management of the animals medical condition. All clinical procedures were done with client (owner) consent. All necropsy specimens were obtained following owner consent for necropsy. Animals either died from clinical complications of their disease (status epilepticus, aspiration pneumonia, cardiorespiratory arrest following herniation), or more commonly following euthanasia at the owner's request due to disease progression and/or poor prognosis. All animals were diagnosed, treated and euthanised as per standard clinical management in accordance with standards established by the American Veterinary Medical Association usually by intravenous overdosage with concentrated barbiturate solution. All samples were taken to either obtain a clinical diagnosis antemortem or post mortem, or in the case of surgical resection, samples were taken during debulking/resection of intracranial masses. Samples of tumor from necropsy were collected within 20 minutes of death and snap frozen in liquid nitrogen. Surgical samples were similarly stored following collection. Samples of adjacent tumor tissue were processed for routine paraffin embedding and histology whenever fresh tissue was collected in liquid nitrogen. Normal brain samples were collected from both necropsy and archival paraffin embedded material. All tumors were graded by a board certified pathologist (RJH) essentially according to the international WHO classification of human tumors of the nervous system [31]. Meningiomas were graded as either grade I (benign), grade II (atypical) or grade III (malignant); astrocytomas were graded as either grade II (diffuse), grade III (anaplastic) or grade IV (glioblastoma multiforme); oligodendroglial tumors were graded as either grade II (oligodendroglioma) or grade III (anaplastic oligodendroglioma). Animal experiments were approved and conducted according to the Wake Forest University Institutional Animal Care and Use Committee (IACUC) protocol A11-117. All human samples were obtained from the archives of the Tumor Bank of the Comprehensive Cancer Center at Wake Forest University/Brain Tumor Bank of the Brain Tumor Center of Excellence while the Wake Forest University Institutional Review Board (IRB) determined that the study was not human subject research; human specimens were handled according to the Wake Forest (IRB) protocol #8427.

### Cell Culture

The canine SDT-3G and GO6A cell lines were derived from spontaneously occurring canine glioblastoma tumors [32]. The human G48a cell line was derived from a human high grade glioma [33]. SDT-3G and GO6A cells were cultured in Dulbecco’s Minimal Essential Eagles Medium, high glucose (Invitrogen/Gibco, Carlsbad, CA.), supplemented to 4825 mg/L sodium bicarbonate (Invitrogen/Gibco, Carlsbad, CA.), with 10% heat inactivated fetal bovine serum (Invitrogen/Gibco, Carlsbad, CA.) at 37°C and 5%CO_2_. The cells were tested to be free from mycoplasma contamination by PCR.

### Quantitative RT-PCR

Total RNA extraction, cDNA preparation and real-time TaqMan PCR were done as previously described [34]. IL-13RA2 PCR primers and probes were designed based on canine sequence data using Primer Express software resulting in a 121-bp product spanning exons 5-6 (Applied Biosystems, Foster City, CA) (forward TTCATTCATTTGGATGTCGGATTCCT; reverse CAGGGTCCACTATCTCAAAATCCT; probe ATGCTGTGCAAACAAG) TaqMan PCR primers for canine housekeeping genes glyceraldehydes-3-phosphate dehydrogenase (GAPDH), ribosomal protein L13A, glycosyltransferase (HPRT1), and glucuronidase beta (GUSB) were used as previously described (33). PCR products were designed to be less than 150 base pairs in length, with either one of the primer pairs or internal probe placed over an exon-exon junction to allow discrimination between cDNA and gDNA. Transcript quantitation was done using the comparative C_T_ method and reported as relative transcription, or the *n*-fold difference relative to the mean value for individual normal cerebral cortex samples (n=15). Tumor samples that had GAPDH C_T_ values weaker than 3 times the average GAPDH C_T_ value were considered low quality cDNA samples and were discarded.

### Monoclonal antibody production

Amino acid peptides spanning the extracellular portion of human IL-13RA2, with 100% homology between human and canine sequences were synthesized as immunogen for the study. The peptides were conjugated to keyhole limpet hemocyanin (KLH) and Balb/c mice were immunized and boosted. The conjugated antigen was injected with Freund's C/IC adjuvant as follows: 1st immunization, 100 μg; 2nd immunization, 50 μg; 3rd immunization, 50 μg; and the final boost, 50 μg per mouse/shot. Generally, four rounds of immunization/boost were needed. 

Titers were measured by ELISA and the most responsive mice were selected for splenocyte fusion. Hybridoma cells were screened by ELISA and the most productive clones were expanded in DMEM with 10% FBS.

### Purification of monoclonal antibodies

Hybridoma cells were grown in UltraDoma Protein Free media (Lonza). Conditioned media from each monoclonal antibody was collected and loaded by FPLC onto a HiTrap Protein A (for IgG2B) or Protein G (IgG1) HP column (GeHealthcare, Piscataway, NJ). Antibody was eluted with 100 mM Sodium Citrate pH 4.3 (Protein A) or 100 mM Glycine HCl pH 2.7 (Protein G). IgM isotype antibodies were purified by S-200 size exclusion chromatography. Briefly, conditioned media from the hybridoma cells was buffer exchanged to PBS and concentrated to a final volume of 1mL. Sample was injected into a calibrated S-200 sepharose column and the IgM antibody was collected in the void volume. The purity of the monoclonal antibodies was verified by SDS- PAGE.

### ELISA Assay

ELISA plates were coated overnight at 4°C with 100 μl/well of 1 μg/μl IL-13RA2-Fc (R&D Systems, Minneapolis, MN) or immunogenic peptide (Genscript Corp). Non-bound protein was removed and the plate blocked with blocking buffer (2% milk/PBS) for one hour at room temperature (RT). Blocking buffer was removed and dilutions of antibody made in blocking buffer was added to the wells and allowed to incubate for one hour at room temperature (RT). Plates were washed with PBS/0.05% Tween and anti-mouse horseradish peroxidase (HRP) secondary antibody was added. After one hour incubation at RT, plates were washed and detection was performed with 2,2’-azino-bis(3-ethylbenzthiazoline-6-sulfonic acid (ABTS). Color was allowed to develop and the plates were read at OD 405 nm.

### SDS-PAGE and Western blot analyses

Cell lysates were prepared from sub confluent cultures. Cells were washed with PBS and lysed in radioimmunoprecipitation assay buffer (PBS, 0.5% sodium deoxycholate, 0.1% SDS, and 0.5% Igepal) containing mammalian protease inhibitor cocktail (Sigma). Nonmalignant brain and pathologist-verified tumor tissue were minced into small pieces while frozen and homogenized in radioimmunoprecipitation assay buffer with mammalian protease inhibitor cocktail. Lysates were passed through an 18-gauge needle to shear the DNA and were incubated on ice for 60 minutes. Nonsoluble debris was pelleted at 10,000 xg for 10 mins and the supernatant was collected and stored at −80°C until use. Additional normal human brain lysates were purchased from Chemicon International and Clontech Laboratories. Lysates were separated by SDS-PAGE using 10% or 15% acrylamide. Proteins were then transferred to a polyvinylidene difluoride membrane (Perkin Elmer, Waltham, MA) and blocked for at least 1 hr with Blotto (5% milk in PBS/0.05% Tween 20). Membranes were incubated with primary antibody diluted in Blotto overnight at 4°C while shaking. β-actin (1:50,000) antibody was purchased from Sigma. Following three 5-min washes in PBS/0.05% Tween 20, membranes were incubated with secondary antibody conjugated with horseradish peroxidase (goat anti-mouse IgG at a dilution of 1:5,000 in Blotto for 1 hr. Membranes were washed three times for 5 min each in PBS/0.05% Tween 20 and detection was done using the Enhanced Chemiluminescence Plus Western Blotting Detection System (GeHealthcare, Piscataway, NJ). Membranes were exposed to autoradiographic film for various times. Films were scanned at 600× dpi and images were compiled using Jasc Paint Shop Pro version 6.0.

### Immunoprecipitation

Cell lysates were prepared from sub confluent cultures. Cells were washed with PBS and lysed in radioimmunoprecipitation assay buffer (PBS, 0.5% sodium deoxycholate, 0.1% SDS, and 0.5% Igepal) containing mammalian protease inhibitor cocktail and 1 mmol/L sodium vanadate. Cell lysate (400 μg) was incubated with 10 μg monoclonal antibody overnight at 4 °C. Twenty microliters of a 50% PBS/bead slurry containing 10 μL packed protein G-Sepharose beads (Sigma) were added and incubated overnight at 4 °C. Beads were collected by centrifugation, washed three times with ice-cold radioimmunoprecipitation assay buffer, and resuspended in 50 μL of 3× SDS sample buffer (New England Biolabs, Ipswich, MA). Samples were heated at 100°C for 5 minutes. Supernatant was collected and stored at −20°C until separated using SDS-PAGE. 

### Immunofluorescent staining

G48a cells, a primary high grade glioma cell line established in this laboratory, were plated and grown overnight on glass slides in RPMI-1640 containing 4 mg glucose/ml and 10% FBS. After 24 hrs, slides were washed in PBS, fixed for 2 min in cold acetone and washed twice in PBS. Slides were washed in PBS for three changes at RT for 5 min each. Cells were blocked for 1 hr in 10% normal goat serum (Invitrogen). Monoclonal antibodies were diluted in PBS/1.5% normal goat serum and incubated overnight at 4 °C. Slides were washed in PBS for three changes at RT for 5 min each. Secondary antibody (Anti-mouse Alexa Fluor-488) was applied and incubated at RT for 1 hr. Nuclei were visualized with DAPI. Slides were washed well in PBS and mounted with FluoreGuard Mounting Media (ScyTek).

### Immunohistochemistry

Biopsy specimens of tumors and normal brain tissues were fixed in 10% formalin and embedded in paraffin. Sections were cut at a thickness of 4-6 μm. Slides were heated at 65°C, de-paraffinized in xylene, and re-hydrated. Antigen retrieval was performed with 10 mM sodium citrate buffer, pH 6.0, by microwaving twice for 5 min. Endogenous peroxidase activity was quenched with Peroxide Blocking Kit (ScyTek Laboratories, Logan, UT). Slides were blocked and incubated with primary antibody or matched isotype control overnight at 4 °C For peptide blocking studies, primary antibody was incubated the 10-fold excess of recombinant peptide for 6 hr prior to application. Slides were washed with PBS followed by incubation with polymer HRP anti-mouse antibody (Vector Labs) for 20 min. Visualization with Impress NovaRed (Vector Labs) was performed and allowed to develop for 4 min. Slides were counterstained with hematoxylin for 2 min, dehydrated and mounted with Permount (Fisher). Photomicrographs were taken with a 20x or on an Olympus IX70 microscope using a Retiga 2000R camera with ImagePro Plus v5.1 software.

### Flow Cytometry

1.5x10^5^ cells were blocked with PBS (pH 7.2)/1% BSA for 1 hr at 4 °C. 15 μg of MAb 1E10B9 or mouse IgG1 (Rockland Immunochemicals, Gilbertsville, PA) was added and the cells were incubated for an additional 2 hr at 4 °C in a volume of 100 μl. Cells were then washed in PBS/1% BSA and resuspended in 100 μL PBS/1% BSA containing 4 μg of anti-mouse Alexa Fluor-488 (Invitrogen, Eugene, OR) or anti-mouse PE (Beckman Coulter Brea, CA) for 1 hr at 4 °C. Samples were washed three times with PBS/BSA before undergoing FLOW analysis on a FACSCalibur or an Accuri6 Flow Cytometer. (BD Biosciences, San Jose, CA). Data were analyzed using Flowing Software V 2.5.0 (Turku, Finland).

### Live Cell Binding

2x10^4^ T98G, U-251 MG (ATCC, Manassas, VA) and G48a human GBM cells were allowed to adhere to glass cover slides overnight at 37 °C with 5% CO_2_ in 500 μL media. 15 μg/milliliter of 1E10B9 mAb or isotype control mouse IgG1 (Rockland Immunochemicals) were added at designated time points. Following incubation, cells were fixed with 250 μL 10% formalin (Fisher Scientific, Kalamazoo, MI) for 15 min at 37 °C. Slides were washed three times with PBS/1% BSA (Fisher Scientific) and incubated with permeabilization buffer (PBS/0.1% Triton X-100 (EMD Chemicals Inc., Darmstadt, Germany) for 10 min at 37 °C. Slides were washed with PBS followed by F-actin staining with Phalloidin-Alexa 488 (Invitrogen) and location of the antibody was detected with anti-mouse alexa-555 (Invitrogen). Nuclei were stained with 4',6-Diamidino-2-Phenylindole dichloride (DAPI; Invitrogen, 1:2000). Cover slips were washed with PBS prior to mounting with FluoreGuard Mounting Media (ScyTek Laboratories, Logan, UT). 

**Figure 1:**
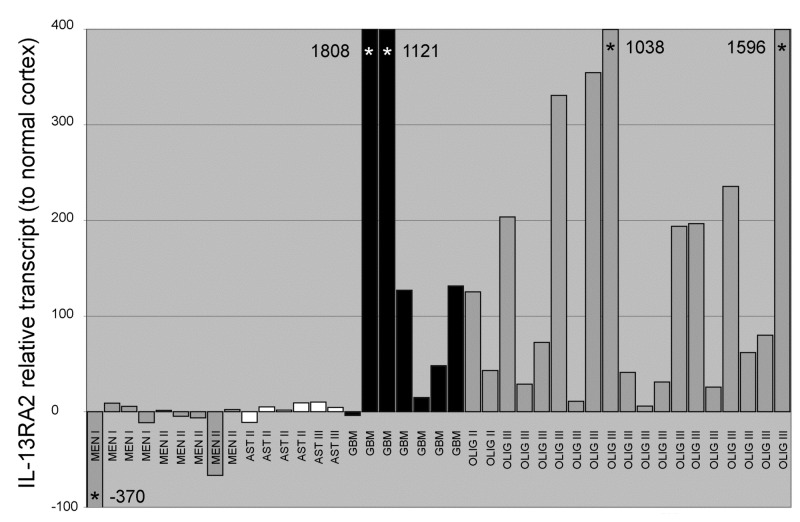
Quantitative TaqMan RT-PCR comparing expression of IL-13RA2 in canine primary brain tumors. Elevated expression, relative to normal canine brain cortex, is seen predominantly in high grade glial tumors, essentially mirroring protein expression determined by western blotting. Off scale values are marked with an asterisk and value. MEN – meningioma; AST – astrocytoma; GBM – glioblastoma multiforme; OLIGO – oligodendroglioma.

### Recombinant Protein Expression and Purification

Canine IL-13 was cloned from published sequences obtained from Genebank (442990). In addition the amino acid corresponding to the 13th residue was mutagenized from Glutamine (E) to a Lysine (K) using Phusion Site Directed Mutagenesis Kit (ThermoFisher). This cytokine was further cloned in-frame to the N-terminal end of a modified *Pseudomonas* exotoxin *A* (PE38QQR) to generate a single chain cytotoxin as previously described for human IL-13 [35]. *E. coli* BL21 (λDE3) cells were transformed with this plasmid and 1.0 liter of LB broth containing 100 μg ampicillin, 4 g glucose, and 0.4 g MgSO_4_/1.0 liter culture was grown until log phase. Protein expression was induced with isopropylthio-β-galactoside (IPTG) for 90 min. Inclusion bodies were isolated, denatured in 8M guanidine solution and renatured in a dithioerythritol and oxidized glutathione reduction-oxidation mixture. After dialysis, recombinant proteins were purified by ion exchange chromatography using FPLC (GE Healthcare Biosciences, Piscataway, NJ). 

### TF-1 proliferation assay

TF-1 cells, a pre-leukemic human B cell line, (ATCC, Manassas, VA) were grown in the presence of increasing concentrations canIL-13.E13K or canIL-13 in 96-well culture plates. After 72 hrs of incubation at 37 °C, the rate of proliferation of the TF-1 cells was determined by a colorimetric MTS [3-(4, 5-dimethylthiazol-2-yl)-5-(3-carboxymethoxyphenyl)-2-(4-sulfophenyl)-2*H*-tetrazolium, inner salt]/PMS (phenazine methosulfate) cell proliferation assay (Promega, Madison, WI).

**Figure 2:**
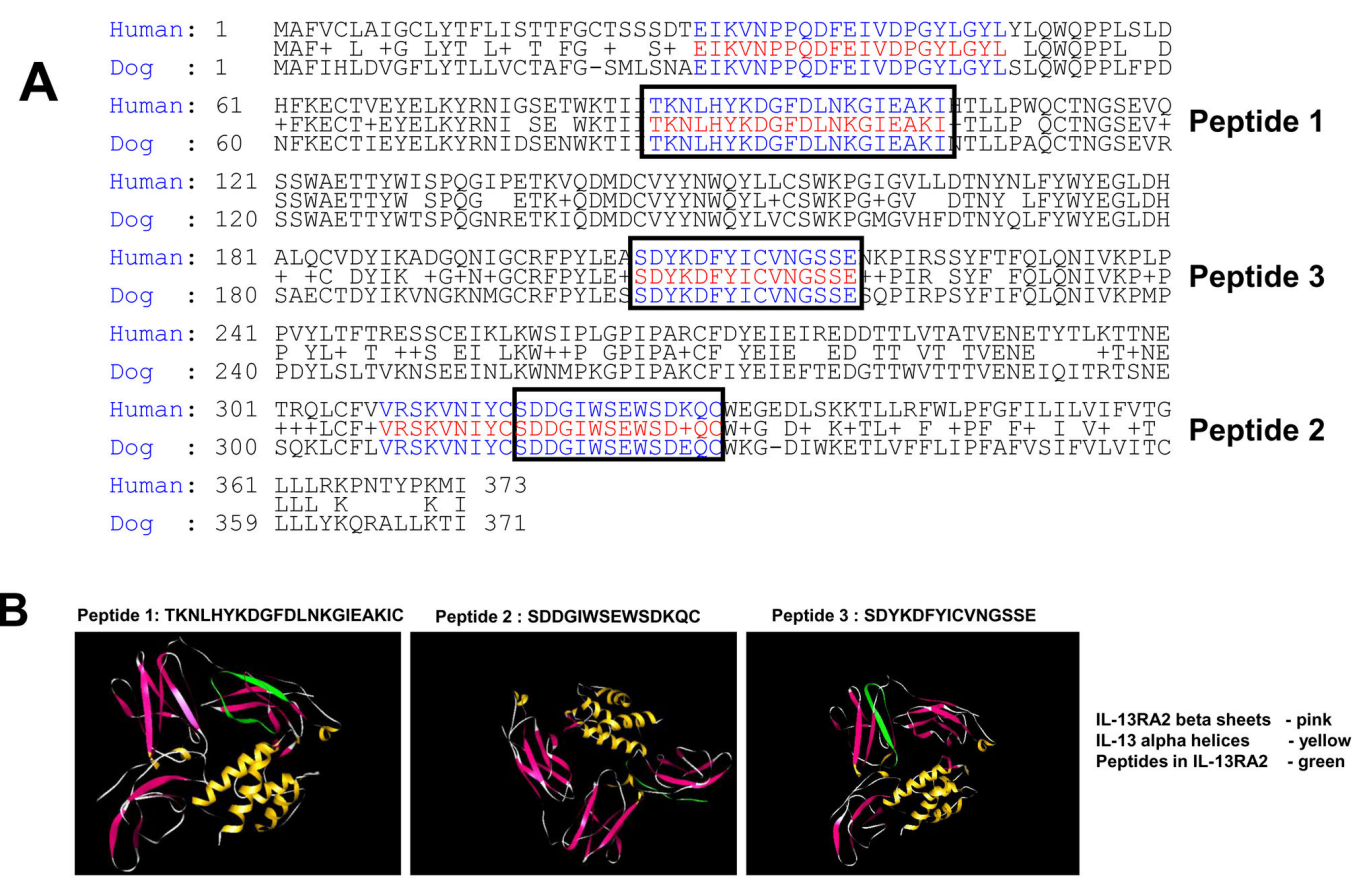
Alignment of human and canine sequences of IL-13RA2. ***A***, The sequences were obtained from the NCBI database. The regions of complete sequence identity between the two species that were utilized as immunogens are boxed. ***B***, Ribbon structure of IL-13RA2 in contact with its natural ligand, IL-13. The three immunogenic peptides used for raising monoclonal antibodies are shown in green. Peptide 1 is located in the extracellular domain of the receptor, Peptide 2 in the vicinity of the ligand binding to the receptor and Peptide 3 is within the extracellular, near transmembrane domain.

### Cytoxicity and Blocking Assay

Human glioblastoma cell line U-251 MG, human primary glioblastoma cells BTCOE 4795 and canine primary glioblastoma cells (GO6-A) were plated into 96-well culture plates and allowed to attach overnight. HuIL-13.E13K or canIL-13.E13K was added to the cells and incubated for 1 h at 37 °C. An equal volume of 0.1% bovine serum albumin in PBS was added to cells for assays without blocking ligand. Increasing concentration of the human or canine IL-13.E13K-PE38QQR was added and the cells were incubated for 48 hrs. Cell viability was determined using the colorimetric MTS/PMS method. Cells treated with high concentrations of cycloheximide served as background for the assay.

### Statistical analysis of RT-PCR data

Data were divided into nominal categories of tumor type, and further divided into ordinal categories of grade. Kruskal-Wallis one way analysis of variance was used to compare the variation in expression of IL-13RA2 mRNA among tumor types, and Jonckheere-Terpstra tests were done to compare variation based on grade within tumor types. When significant differences were evident, Mann-Whitney tests were used for pair-wise comparisons. A sequentially rejective modification of Bonferroni’s multiple comparison adjustment was used to confirm significant results. An unpaired t-test was used in cell viability assays. Statistical significance was defined as P≤0.05.

**Table 1:** Properties of monoclonal antibodies raised against three antigenic peptides.

**Cell line**	**Isotype**	**ELISA (peptide)**	**ELISA (IL-13RA2-Fc)**	**Western Blot - Lysates**	**Western Blot IL-13RA2-FC**	**IF**	**IHC**	**Flow cytometry**	**Live cell binding**
**Peptide 1**									
**2G12C3**	**IgG2β, κ**	+*	**+**	**+**	**+**	-	-	-	-
**2G12E2**	**IgG2β, κ**	**+**	**+**	**+**	**+**	ND	ND	ND	ND
**4C3A8**	**IgG2β, κ**	**+**	**+**	**+**	**+**	ND	ND	ND	ND
**4C3B7**	**IgG2β, κ**	**+**	**+**	**+**	**+**	ND	ND	ND	ND
**Peptide 2**									
**6D3E9**	**IgM,κ**	**+**	**+**	**+**	**+**	**+**	**+/-**	-	ND
**6D3E3**	**IgM,κ**	-	-	ND	-	ND	ND	ND	ND
**3D4E9**	**IgG1,κ**	**+/-**	-	ND	-	ND	ND	ND	ND
**3D4G10**	**IgG1,κ**	**+**	-	-	**+**	-	ND	ND	ND
**6F6B3**	**IgG1,κ**	**+**	**+**	**+**	**+**	-	ND	-	-
**6F6C2**	**IgG1,κ**	**+**	**+**	**+**	**+**	ND	ND	ND	ND
**4G9G3**	**IgG1,κ**	**+**	**+/-**	ND	**+/-**	-	ND	ND	ND
**4G9H4**	**IgG1,κ**	**+**	-	ND	-	ND	ND	ND	ND
**Peptide 3**									
**1E10B9**	**IgG1,κ**	**+**	**+**	**+**	ND	+	+	+	+
**1E10F9**	**IgG1,κ**	**+**	**+**	**+**	ND	ND	ND	ND	ND
**3D11E11**	**IgG1,κ**	**+**	-	-	ND	ND	ND	ND	ND
**3D11H7**	**IgG1,κ**	**+**	-	-	ND	ND	ND	ND	ND
**5F3D7**	**IgG1,κ**	**+**	-	-	ND	ND	ND	ND	ND
**5F3G10**	**IgG1,κ**	**+**	-	-	ND	ND	ND	ND	ND

* + strongly positive; - negative; +/-, positive; and ND, not performed

## Results

### Gene expression of IL-13RA2 in primary canine brain tumors

In expectation that the similarities between human and canine primary brain tumors exist at the genetic/molecular level, we preliminarily screened 40 canine archival tumor specimens for *IL-13RA2* gene expression (Figure 1). A total of 15 samples of normal cerebral cortex (4 frozen and 11 paraffin embedded samples) and 40 tumor samples were analyzed (11 frozen and 29 paraffin embedded samples). Of these samples, there were 9 meningiomas (4 Grade I, 5 atypical Grade II); 13 astrocytomas (4 grade II, 1 grade III, 7 GBM) and 19 oligodendrogliomas (3 grade II, 16 Grade III). For all genes analyzed, the averages of normalized values and the standard deviations for both frozen and paraffin embedded samples were not significantly different. Increased expression was seen predominantly in the high grade gliomas (Figure 1). Median values for expression relative to normal canine cortex were -4.7 fold (meningiomas); 5.1 fold (astrocytomas), 127 fold (GBM) and 133 fold (oligodendrogliomas grade III). Differences in expression based on grade were present within the astrocytic tumors, with the highest expression seen in GBMs (p=0.003). Expression in high grade oligodendrogliomas was significantly greater than both meningiomas (grade I and II combined; p<0.0001) and lower grade astrocytomas (grade II and III combined; p=0.0004). Expression in GBMs was also significantly greater than meningiomas (grade I and II combined; p=0.002). There was no significant difference between expression in high grade oligodendrogliomas and GBMs (p=0.97). Thus, we obtained evidence for the over-expression of *IL-13RA2* mRNA over-expression in various canine tumors similarly to humans [36]. 

**Figure 3:**
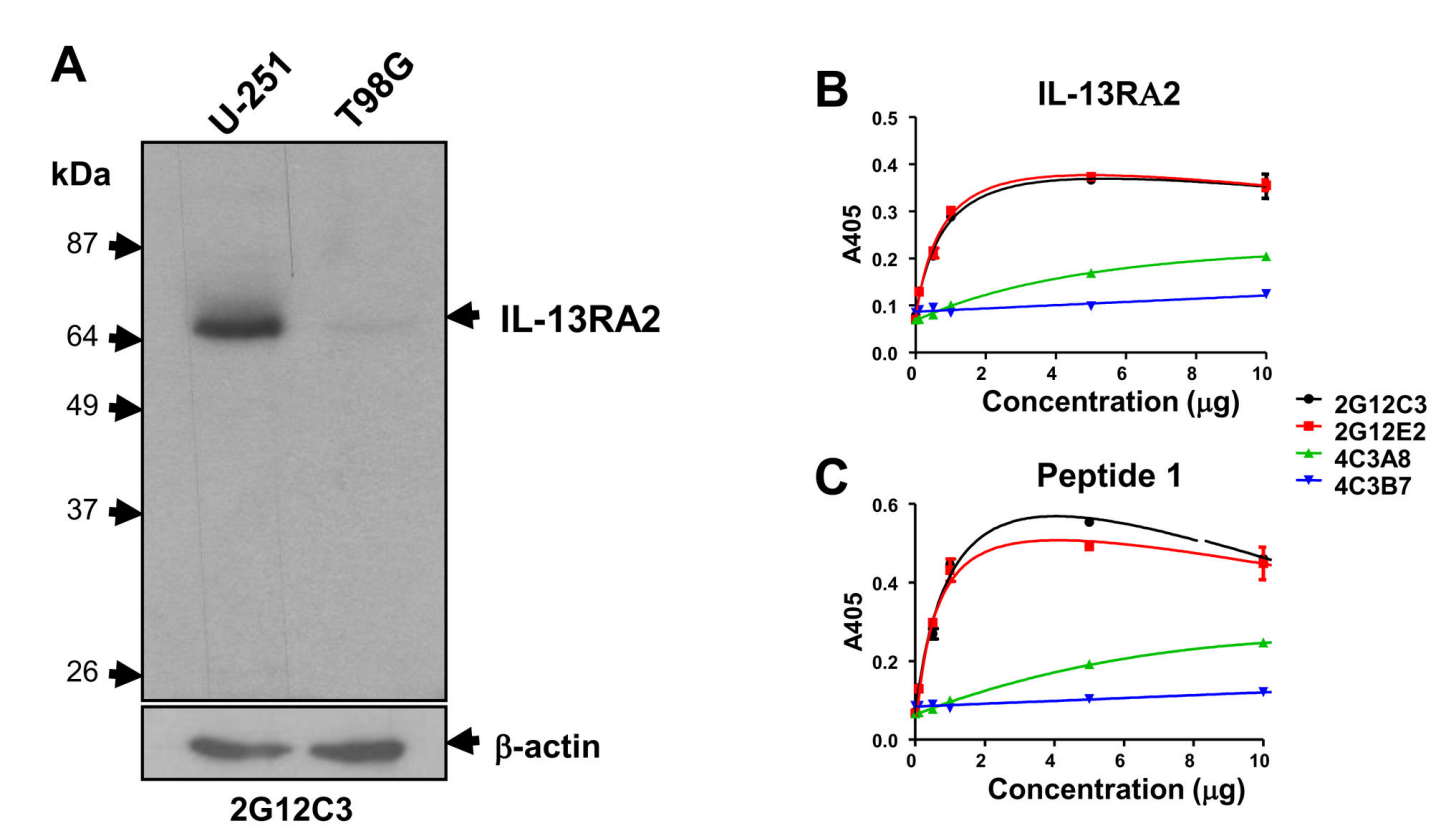
Immunoreactivity of monoclonal antibodies induced by Peptide 1 and recognition of synthetic and recombinant immunogens. ***A***, Western blot of U-251 MG and T98G human GBM cell lysates using media of 3G12C3 hybridoma cells. ELISA was conducted using either recombinant IL-13RA2-Fc, ***B*** or the synthetic Peptide 1, ***C***.

**Figure 4:**
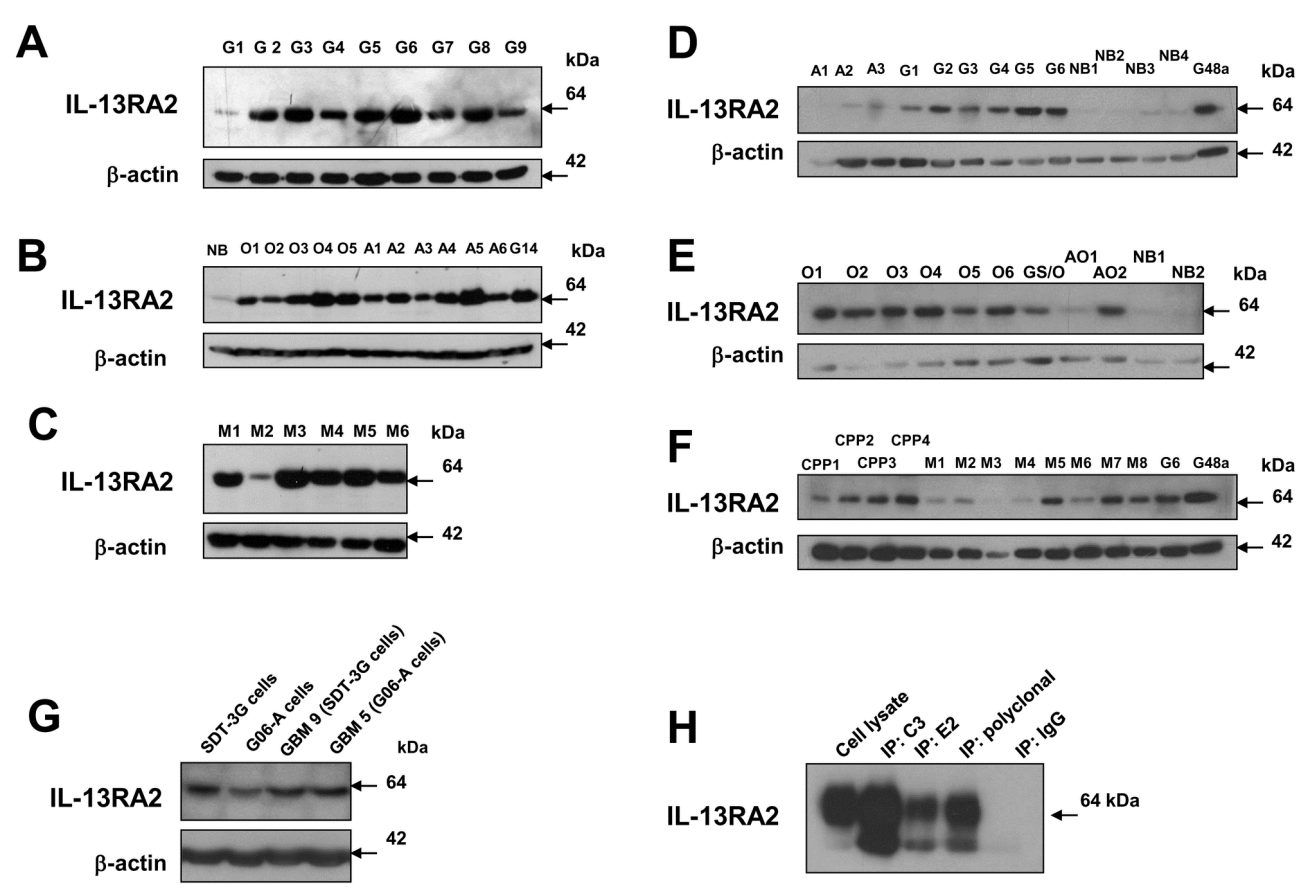
Immunoreactive IL-13RA2 in human and canine brain tumor specimens/cells by purified MAb’s of Peptide 1 ***A***, Human glioblastoma (G), ***B***, oligodendroglioma (O), astrocytoma (A), normal brain (NB), and G14 human GBM tumor lysate; meningioma (M), ***C***, tissue lysates immunoreactivity using Western blots. Canine astrocytoma, glioblastoma and normal brain, ***D***; oligodendroglioma, gliosarcoma (GSO) and mixed astro-oligo (AO), ***E***; and choroid plexus papilloma (CPP) and meningioma, ***F*** tissue lysates immunoreactivity using western blots. Western blot of cell lines and parent tumor tissue obtained from dogs with spontaneous GBM, ***G***. Immunoprecipitation of IL-13RA2 from U-251 MG cell lysate using either MAb 2G12C3, MAb 2G12E2 or a polyclonal antibody (R&D Systems #AF146); the polyclonal antibody was used for the receptor detection after immunoprecipitation***, H***.

### MAbs raised against Peptide 1 of homology region between human and canine IL-13RA2

To verify the exact expression profile of IL13RA2 in various primary brain tumors, and validate the canine translational model we generated bi-species specific antibodies against IL-13RA2 against three different regions of the receptor with 100% sequence identity (Figure 2) (Table 1). Two hybridomas with two subclones of each hybridoma (2G12C3, 2G12E2, and 4C3A8, 4C3B7) were obtained for antibodies raised against Peptide 1 (TKNLHYKDGFDLNKGIEAKIC) (Figure 2) in the immunization and selection procedure. High concentrations of purified antibody were obtained from all clones (not shown). Antibodies were assayed by ELISA using either recombinant IL-13RA2 or antigenic Peptide 1 (Figure 3A and 3B, respectively). MAb 2G12C3 and 2G12E2 strongly reacted with immunogens while MAb obtained from other subclones, 4C3A8 and 4C3B7, demonstrated significantly less reactivity (Figure 3A-B).

**Figure 5:**
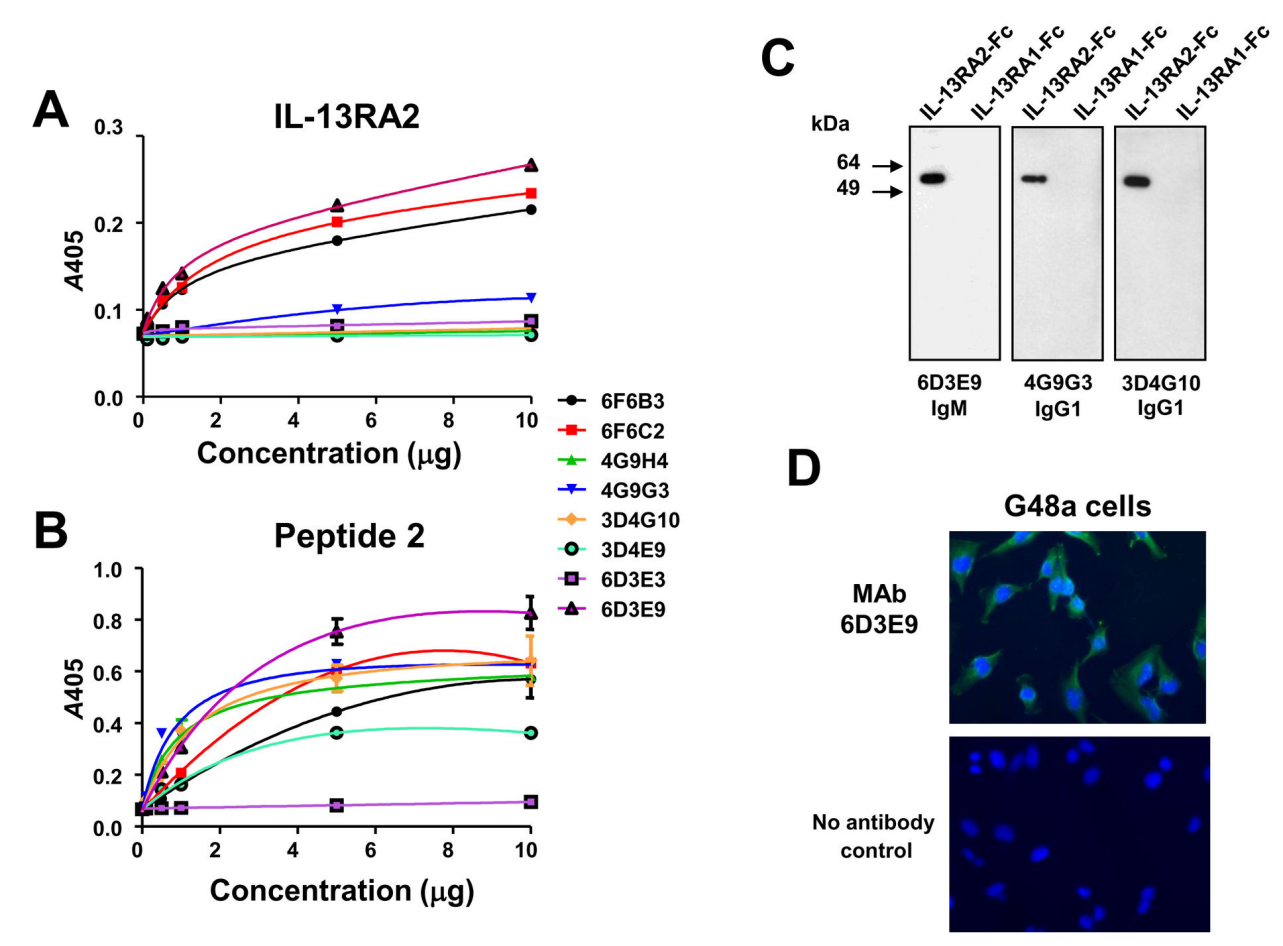
Imunoreactivity of MAbs obtained with immunization using Peptide 2. Reactivity of MAbs raised against Peptide 2 in ELISA using recombinant IL-13RA2-Fc, ***A***; and Peptide 2, ***B***. Detection of recombinant IL-13RA2, but not of IL-13RA1-Fc with MAbs 6D3E9, 4G9G3 and 3D4G10, The proteins were loaded at 0.5 µg/lane. ***C***. Immunofluorescence in G48a cells using MAb 6D3E9, ***D***.

Subclone 2G12C3 was selected for further characterization. MAb 2G12C3 showed asingle immunoreactive band (consistent with IL-13RA2) on western blots of cell lysates of high expressing (U-251) and low expressing (T98G) GBM cells (Figure 3C) comparable to previous reports [7]. Western blots of tissue lysates from human brain tumors showed a single band of variable intensity, from very low to very high in 9/9 GBMs (Figure 4A), 5/5 oligodendrogliomas and 6/6 astrocytomas, with minimal signal in normal brain (Figure 4B). Five out of six meningioma tissue lysates showed strong signal, consistent with previous gene expression data (Figure 4C) [33].

Parallel western studies were done using canine brain tumor lysates of GBM, astrocytoma (II), oligodendroglioma (III), mixed oligodendroglioma/astrocyoma, choroid plexus papilloma, meningioma and normal brain (Figures 4D-F). All canine GBMs (G) contained immunoreactive IL-13RA2 similarly to human specimens while canine astrocytomas (A) seem to express less of the receptor than the human tissue lysates (Figure 4D). Oligodendrogliomas (O) expressed the receptor in a majority of samples (8/9) and at a level similar to human specimens (Figure 4E). Interestingly, four specimens of canine choroid plexus papilloma samples (CPP) were noticeably enriched in IL-13RA2 (Figure 4F). Moreover, canine meningiomas (M) expressed readily detectable receptor (6/8), but less than in humans (Figure 4F). Normal canine brain samples either did not contain immunoreactive receptor or showed negligible amounts (Figure 4D-E).

**Figure 6:**
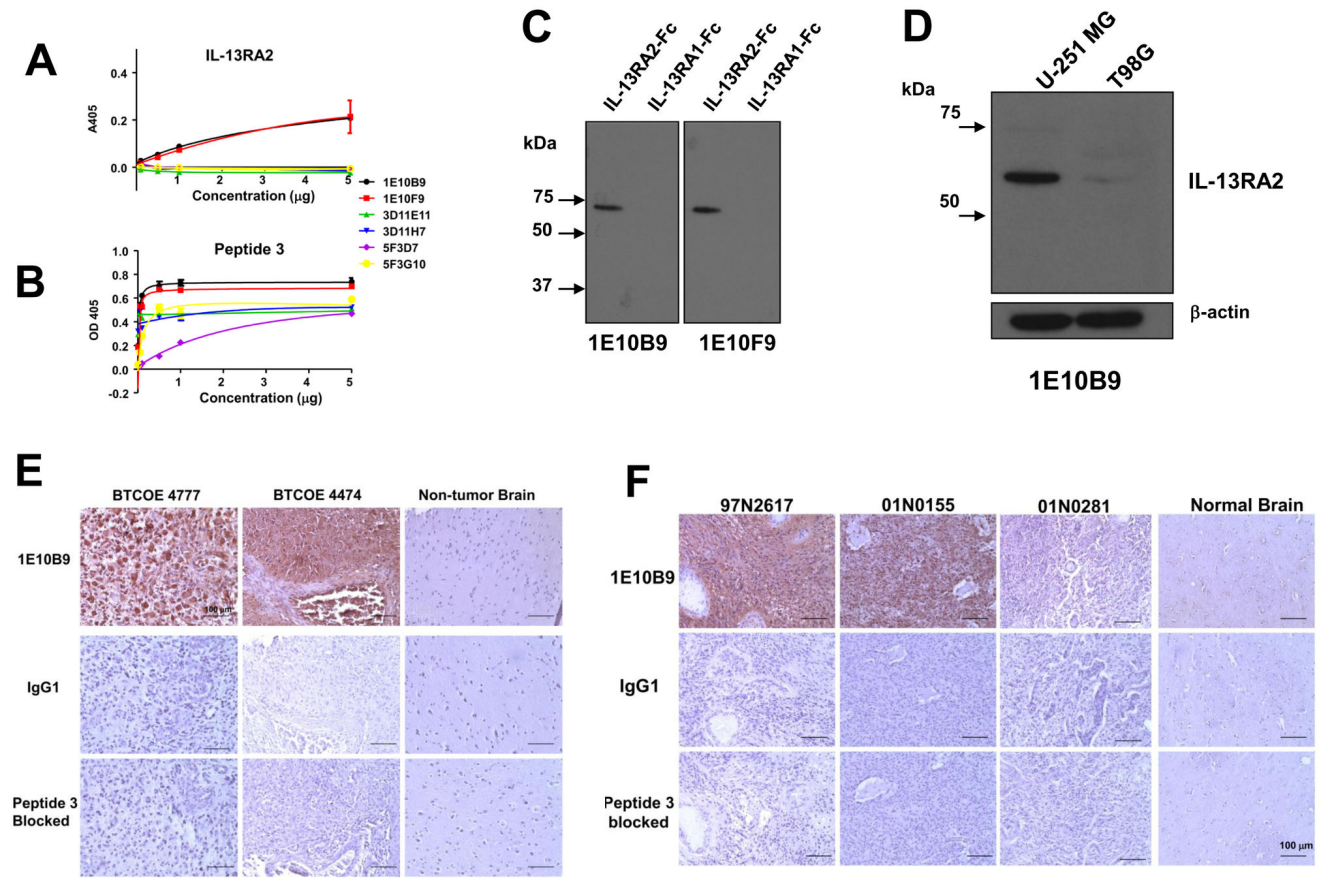
Imunoreactivity of MAbs obtained with immunization using Peptide 3. Reactivity of MAbs raised against Peptide 3 in ELISA using recombinant IL-13RA2-Fc, ***A***; and the synthetic Peptide 3, ***B***. Detection of IL-13RA2, but not of IL-13RA1-Fc with MAbs 1E10B9 and 1E10F9, ***C***. Immunoreactive IL-13RA2 in Western blot of U-251 MG and T98G cell lysates using MAb 1E10B9, ***D***. Expression of IL-13RA2 detected by immunohistochemistry using MAb 1E10B9 in human GBM specimens and normal brain, ***E***, and canine GBMs and normal brain, ***F***.

We also examined immunoreactivity of IL-13RA2 in the lysates of canine GBM cell lines, SDT-3G and G06-A cells, together with their matching tissue specimens (Figure 4G). The expression of the receptor was detected in tissue lysates and was retained by the corresponding cells in culture. Immunoreactive IL-13RA2 was retrieved using immuoprecipitation assays utilizing lysates of U-251 MG human established GBM cells and the 2G12C3 and 2G12E2 MAbs and a commercially available polyclonal antibody (R&D Systems # AF146) (Figure 4H). None of the MAbs raised against Peptide 1 could be successfully utilized for immunohistochemical staining or Flow Cytometry (not shown).

### MAbs raised against Peptide 2 of homology region between human and canine IL-13RA2

Four hybridoma clones with two subclones each were obtained using Peptide 2 (SDDGIWSEWSDKQC) as immunogen (Figure 2) (Table 1). Three of the clones were IgGs and one was of the IgM class. The purified MAbs of 6F6C2, 6F6B3 and 6D3E9 reacted strongly with recombinant IL-13RA2 and Peptide 2 in ELISA; only the MAb 6D3E3 did not react with either the receptor or peptide at all (Figure 5A-B). We further tested these antibodies in western blots using recombinant IL-13RA2 and IL-13RA1 as a control. 6D3E9 IgM, 4G9G3 and 3D4G10 IgG1s reacted strongly with recombinant IL-13RA2 but not with IL-13RA1 (Figure 5C). Some immunofluorescent staining of G48a human GBM cells (high IL-13RA2 expressors) using the IgM MAb (6D3E9) was demonstrated(Figure 5D). Immunofluorescent staining was not obtained with any of the IgG_1_s of this group of MAbs, and consistent results were not obtained for any antibodies using immunohistochemical staining of formalin fixed tissue or flow cytometry (not shown).

**Figure 7:**
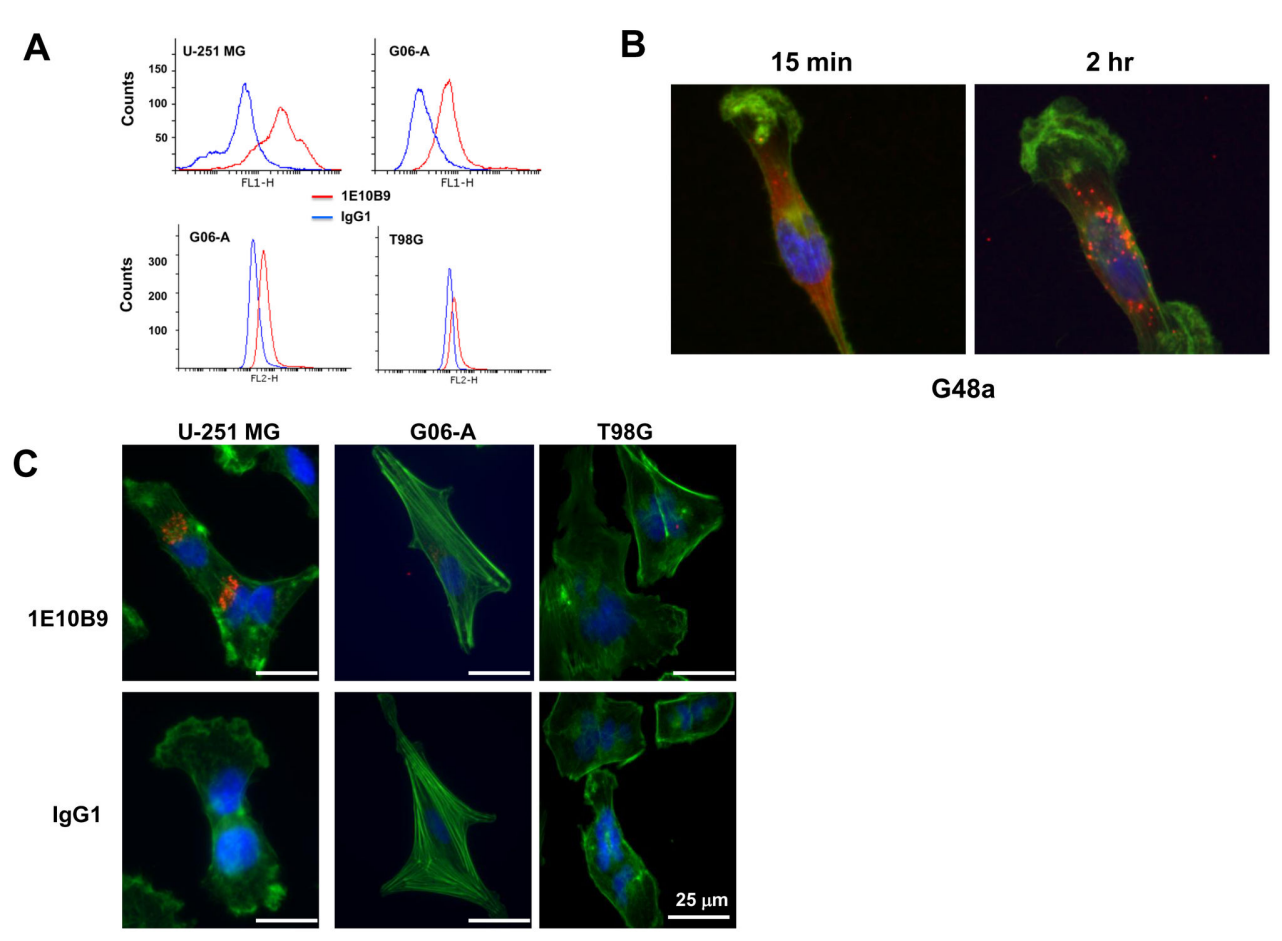
MAb 1E10B9 binds to live GBM cells. Flow cytometry on human U-251 MG cells, canine GBM G06-A cells, and human T98G cells using MAb 1E10B9, ***A.*** Internalization of MAb 1E10B9 by G48a human GBM cells, ***B.*** Internalization of MAb 1E10B9 by U-251, G06-A, and T98G cells after 4-hr incubation, ***C***.

### MAbs raised against Peptide 3 of homology region between human and canine IL-13RA2

In the last immunization protocol using Peptide 3 (SDYKDFYICVNGSSE) (Figure 2) three hybridoma subclones were obtained with two subclones each (Table 1). The MAbs were purified from the media and demonstrated variable reactivity by ELISA using either recombinant receptor or immunogenic Peptide 3 (Figure 6A-B). Two IgG MAbs, 1E10B9 and 1E10F9 reacted most positively in these assays and they were further characterized in additional experiments. In western blots with recombinant IL-13RA2 and IL-13RA1, these antibodies reacted only with the tumor-associated receptor, IL-13RA2 (Figure 6C). MAb 1E10B9 also detected an immunoreactive band of IL-13RA2 in U-251 MG and T89G cells (Figure 6D), similar to Peptide 1 clone 2G12C3 (Figure 3C).

We next performed immunohistochemistry on human and canine tumor specimens. MAb 1E10B9 demonstrated strong staining in human and canine tissue specimens of GBM xenograft tumors (Figure 6E-F). The binding was specific and not detected to any significant degree in normal brains or tumors with very low level of the receptor like 01N0281 (Figure 6F). The staining was absent on vascular components of tumors (Figure 6E-F).Additionally, MAb 1E10B9 using flow cytometry on human U-251 MG and T98G cells and canine G06-A cells (Figure 7A) was performed. We found robust binding of the antibody to the receptor present on the surface of live cells according to the expected levels of the receptor expression on the studied cells. We also tested whether MAb 1E10B9 can bind the receptor and induce its internalization. This antibody was uptaken by G48a GBM cells and after two hrs of incubation it was found mostly in the perinuclear region (Figure 7B), similarly to other polypeptides binding IL-13RA2 [15]. The internalization of the antibody was also seen after 4 hrs of incubation in U-251 MG human GBM cells and G06-A canine GBM cells, but little or not at all in T98G human GBM cells that express low levels of the receptor (Figure 7C). Thus, MAb 1E10B9 recognizes IL-13RA2 in ELISA, western blot and immunohistochemistry assays, binds to live cells expressing the receptor and induces the receptor internalization.

**Figure 8:**
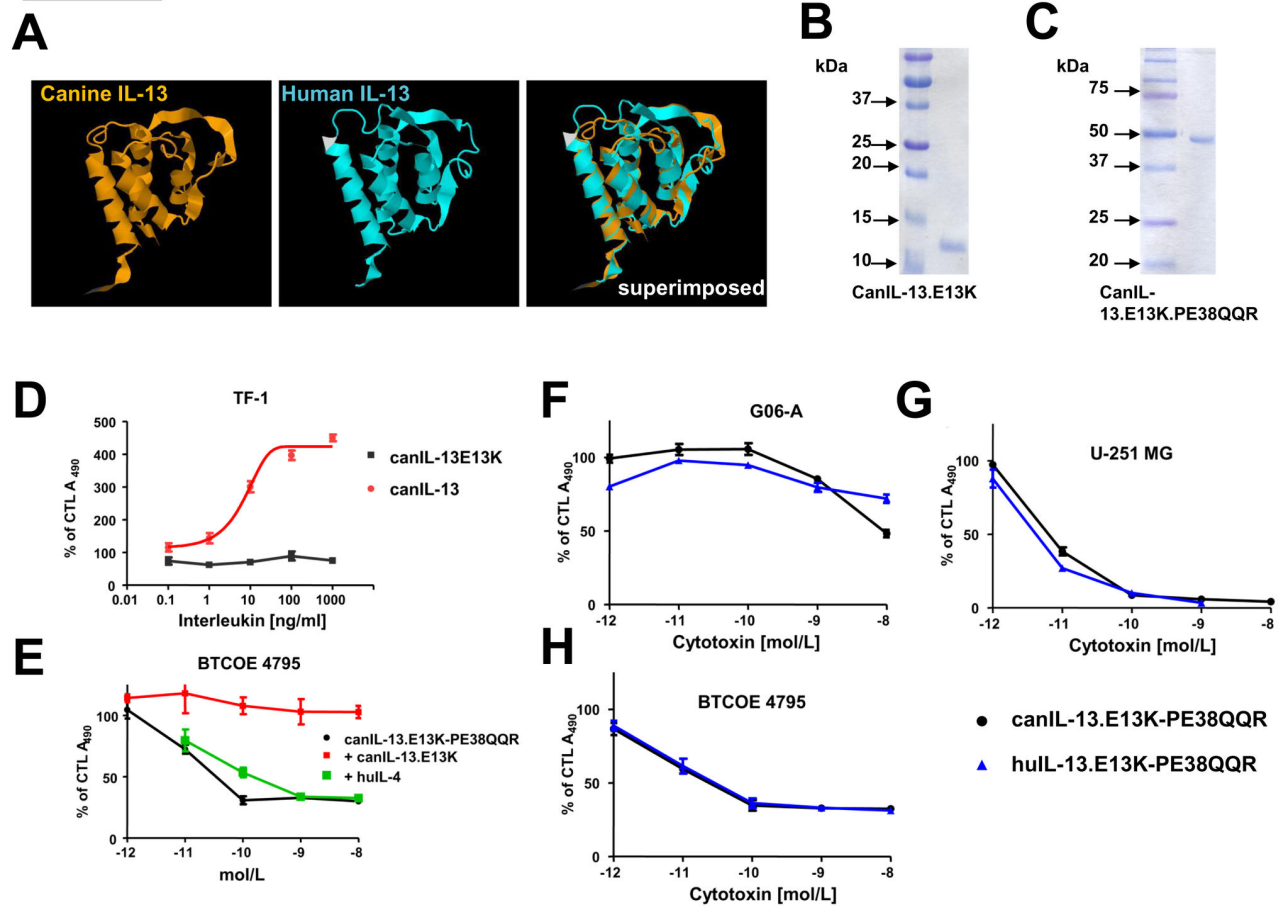
Production and testing of canIL-13.E13K and canIL-13.E13K based cytotoxin. Superimposition of canIL-13 and huIL-13 molecules, (3D reconstruction using JMol), ***A.*** Purified canIL-13.E13K and canIL-13.E13K cytotoxin, (10% SDS-PAGE), ***B and C***. Activation of TF-1 cells proliferation by cytokines, ***D***. Cytotoxicity of canIL-13 cytotoxin and its neutralization on BTCOE 4795 human GBM cells, ***E***. P<0.015 and <0.007 for differences between the cytokines (in ***D***) and the cytotoxin killing vs. neutralization with canIL-13.E13K alone (in ***E***) using an unpaired t-test. Cytotoxicity of canIL-13.E13K cytotoxin on canine GBM G06-A cells, ***F***. Cytotoxicity of canIL-13.E13K cytotoxin on human GBM established (U-251 MG), ***G***, and low passage human GBM cells (BTCOE 4795), ***H***. CTL – control. Vertical bars represent SEM and if not seen, they are smaller than the points.

### Canine IL-13 Based Cytotoxins Kill GBM Cells in a Targeted Manner

Human and canine IL-13 have very similar 3-D structure as seen in Figure 8A, but there are clear variances in spatial arrangements of these molecules most likely related to the fit of the ligands to their species-relevant receptors. Previous studies (not shown) determined that human IL-13 (huIL-13) conjugated toxins did not result in efficient targeted killing of canine glioma cell lines *in vitro*. Therefore, we cloned and produced highly purified recombinant canine IL-13.E13K (canIL-13.E13K) and a single-chain cytotoxin containing canIL-13.E13K and a derivative of *Pseudomonas* exotoxin A (PE), PE38QQR (Figure 8B-C). This mutant form of IL-13 recognizes IL-13RA2 differentially form the IL-13RA1 [15]. Functionality of canIL-13.E13K was determined by assessing induction of proliferation in human-derived TF-1 cells that express the IL-4RA/IL-13RA1 physiological receptor for IL-13. CanIL-13.E13K was compeltley inactive on these cells while the wild type canIL-13 exhibited prominent profilerative activity (Figure 8D). Thus our recombinant canIL-13 demonstrates biological activity comparable to the human IL-13 cytokine, which can be abrogated by a single amino acid substitution. Next, we tested the ability of canIL-13.E13K to neutralize the cytotoxic action of canIL-13.E13K-PE38QQR on human GBM BTCOE 4795 explant cells that over-express IL-13RA2. CanIL-13 blocked the killing effect of the cytotoxin (Figure 8E) indicating an efficient competition of canIL-13 for human IL-13RA2, unlike huIL-4. 

We next tested the recombinant canIL.E13K-13-PE38QQR cytotoxin on both human and canine GBM cell lines. CanIL-13.E13K-PE38QQR was potent in killing both canine (GO6-A) (Figure 8H) and human (U-251 and BTCOE 4795) GBM cells (Figure 8F and 8G, respectively).

## Discussion

We have generated a panel of monoclonal antibodies against IL-13RA2, a tumor-associated receptor, that are suitable for pharmaceutical targeting (Table 1). These antibodies cross-react with the homologous canine receptor and thus may be used in these species in translational studies. Three various regions of the receptor targeting either extracellular, or ligand-binding domains with 100% of homology between human and canine receptors were chosen for the production of immunogenic peptides, Peptides 1-3 from different regions of the receptor. Most purified antibodies reacted with the immunogenic peptides and recombinant receptor *in vitro*. Also most antibodies were useful in the detection of immunoreactive IL-13RA2 in cell and tissue lysates using western blotting with no cross-reactivity for the closely related IL-13RA1. One antibody raised against Peptide 3 (MAb 1E10B9) was found uniquely to detect the receptor in tissue specimens in situ and in western blots, efficiently bind live cells and be internalized upon binding to the receptor. Our novel antibodies (Table 1) detected IL-13RA2 in a variety of human and canine brain tumors and cell lines on western blots, defining an extended spectrum of potential target tumors beyond the high grade astrocytomas previously reported [3,19]. Importantly, absence or negligible expression of the target was confirmed in both human and canine normal brain.

The presence of the receptor in various tumors other than human and canine GBM tumors differed. For example, human astrocytomas, oligodendrogliomas and meningiomas demonstrated high levels of IL-13RA2 immunoreactivity. While canine oligodendrogliomas showed similarly high presence of the receptor protein, meningiomas were less enriched in the receptor comparatively to human samples. Interestingly, canine choroid-plexus tumors contained high amounts of immunoreactive IL-13RA2; human choroid plexus tumors were not investigated in the current study and further examinaion is warranted. 

One of the isolated antibodies, MAb 1E10B9, demonstrated a number of attractive and unique features. It recognized IL-13RA2 in cell and tumor lysates using western blot and resulted in robust immunohistochemical staining in archival paraffin embedded specimens. Importantly, it bound live GBM cells of both human and canine origin and it was shown to be internalized upon binding to the receptor. This versatility offers multiple potential applications for MAb 1E10B9 for diagnostic, imaging and therapeutic approaches. A recent report showed therapeutic utility of an unconjugated antibody against human IL-13RA2 [37].

IL-13RA2 is an attractive molecular target in a variety of human malignancies and in primary brain tumors in particular. The current study demonstrates that it is also a valid target in a clinically relevant spontaneous animal model of human disease, namely spontaneously occurring canine brain tumors. The IL-13RA2 receptor belongs to a tri-molecular signature of human GBM also including the EphA2 receptor and a transcription factor Fra-1 (19), and recent studies are suggestive of IL-13RA2 and EphA2 belonging to a group of factors characterizing glioma stem-like cells [38-40]. The availability of specific and sensitive antibodies recognizing the receptor under various conditions will be important in further studies examining the pathophysiological role of IL-13RA2 in brain tumors, including its closest translational model in a form of spontaneous canine tumors. Validation of IL-13RA2 as a target in canine brain tumors and generation of a novel canIL-13 based cytotoxin demonstrating potent and specific killing of canine GBM cells will allow for validation and development of IL-13RA2 targeted therapeutic strategies in a clinically relevant translational model system. It is hoped that use of this approach will help bridge the gap between in vitro and rodent based proof of principal experiments and the clinical arena. The model is a large animal model allowing on performing medical procedures in a similar manner to humans with potential benefit to canine patients as well. It is also a model of spontaneously arising brain tumors with pathological, morphological, and molecular features of human tumors and hence giving substantial advantage over any other brain tumor model. 

Previously, the first generation of IL-13 cytotoxin prolonged significantly the survival of patients with recurrent GBM when used in centers experienced with loco-regional deliveries drugs [41], but imaging was not employed and thus drug delivery and its monitoring was not standardized among all centers. Based on the observations presented here, a Phase I clinical trial in the treatment of canine astrocytomas has begun utilizing IL-13RA2- and EphA2-[19] targeted cytotoxins in combination. This trial involves using dogs/real time imaging to define appropriate delivery of the targeted therapy to the “target”. 

## References

[1] Stupp R, Mason WP, van den Bent MJ, Weller M, Fisher B et al. (2005) Radiotherapy plus concomitant and adjuvant temozolomide for glioblastoma. N Engl J Med 352: 987-996. doi:10.1056/NEJMoa043330. PubMed: 15758009. 15758009

[2] Debinski W, Obiri NI, Powers SK, Pastan I, Puri RK (1995) Human glioma cells overexpress receptor for interleukin 13 and are extremely sensitive to a novel chimeric protein composed of interleukin 13 and Pseudomonas exotoxin. Clin Cancer Res 1: 1253-1258. PubMed: 9815919. 9815919

[3] Debinski W, Gibo DM, Hulet SW, Connor JR, Gillespie GY (1999) Receptor for interleukin 13 is a marker and therapeutic target for human high grade gliomas. Clin Cancer Res 5: 985-990. PubMed: 10353730. 10353730

[4] Mintz A, Gibo DM, Webb (Slagle) B, Debinski W (2002) IL13Rα2 is a glioma-restricted receptor for IL13. Neoplasia 4: 388-399

[5] Debinski W (1998) An immune regulatory cytokine receptor and glioblastoma multiforme: an unexpected link. Crit Rev Oncog 9: 255-268. PubMed: 10201630. 10201630

[6] Debinski W, Gibo DM (2000) Molecular expression analysis of restrictive receptor for interleukin 13, a brain tumor-associated cancer/testis antigen. Mol Med 6: 440-449. PubMed: 10952023. 10952023 PMC1949955

[7] Hu N, Gibo DM, Debinski W ( 2005) Cytokine up-regulation of IL-13Rα2 in GBM cells leads to an increased potency of recombinant IL13 cytotoxin. Cancer Therapy 3: 531-542.

[8] Lal A, Glazer CA, Martinson HM, Friedman HS, Archer GE et al. (2002) Mutant epidermal growth factor receptor up-regulates molecular effectors of tumor invasion. Cancer Res 62: 3335-3339. PubMed: 12067969. 12067969

[9] Mintz A, Debinski W (2000) Cancer genetics/epigenetics and the X chromosome: Possible new links for malignant glioma pathogenesis and immune-based therapies. Critic Rev Oncogen 11: 77-95 10795628

[10] Okano F, Storkus WJ, Chambers WH, Pollack IF, Okada H (2002) Identification of a novel HLA-A*0201-restricted, cytotoxic T lymphocyte epitope in a human glioma-associated antigen, interleukin 13 receptor α2 chain. Clin Cancer Res 8: 2851-2855. PubMed: 12231526. 12231526

[11] Mintz A, Gibo DM, Madhankumar AB, Cladel NM, Christensen ND et al. (2008) Protein and DNA-based active immunotherapy targeting interleukin 13 receptor alpha 2. Cancer Biother Radiopharm 23: 581-589. doi:10.1089/cbr.2008.0462. PubMed: 18976118. 18976118 PMC2936944

[12] Zhou G, Ye G-J, Debinski W, Roizman B (2002) Genetic engineering of a herpes virus 1 vector dependent on the IL-13Rα2 receptor for entry into cells: interaction of glycoprotein D with its receptors is independent of the fusion of the envelope and the plasma membrane. Proc Natl Acad Sci USA 99: 15124-15129. doi:10.1073/pnas.232588699. PubMed: 12417744. 12417744 PMC137554

[13] Kahlon KS, Brown C, Cooper LJN, Raubitschek A, Forman SJ et al. (2004) Specific recognition and killing of glioblastoma multiforme by interleukin 13-zetakine redirected cytolytic T cells. Cancer Res 64: 9160-9167. doi:10.1158/0008-5472.CAN-04-0454. PubMed: 15604287. 15604287

[14] Chunbin L, Hall WA, Jin N, Todhunter DA, Panoskaltis-Mortari A et al. (2002) Targeting glioblastoma multiforme with an IL-13 / diphtheria toxin fusion protein in vitro and in vivo in nude mice. Prot Engin 15: 419-427. doi:10.1093/protein/15.5.419. 12034862

[15] Debinski W, Gibo DM, Kealiher A, Puri RK (1998) Novel anti-brain tumor cytotoxins specific for cancer cells. Nat Biotechnol 16: 449-453. doi:10.1038/nbt0598-449. PubMed: 9592393. 9592393

[16] Mintz A, Gibo DM, Madhankumar AB, Debinski W (2003) Molecular targeting with recombinant cytotoxins of interleukin-13 receptor alpha-2-expressing glioma. J Neurooncol 64: 117-123. doi:10.1023/A:1024918916984. PubMed: 12952292. 12952292

[17] Ulasov IV, Tyler MA, Han Y, Glasgow JN, Lesniak MS (2007) Novel recombinant adenoviral vector that targets the interleukin-13 receptor alpha2 chain permits effective gene transfer to malignant glioma . Hum Gene Ther 18: 118-129. doi:10.1089/hum.2006.146. PubMed: 17328684. 17328684

[18] Candolfi M, Xiong W, Yagiz K, Liu C, Muhammad AK et al. (2010) Gene therapy-mediated delivery of targeted cytotoxins for glioma therapeutics . Proc Natl Acad Sci USA 107: 20021-20026. doi:10.1073/pnas.1008261107. PubMed: 21030678. 21030678 PMC2993419

[19] Wykosky J, Gibo DM, Stanton C, Debinski W (2008) IL-13 Receptor alpha-2, EphA2, and Fra-1 as molecular denominators of high-grade astrocytomas and specific targets for combinatorial therapy. Clin Cancer Res 14: 199-208. doi:10.1158/1078-0432.CCR-07-1990. PubMed: 18172271. 18172271

[20] Fankhauser R, Luginbühl H, McGrath JT (1974) Tumours of the nervous system. Bull World Health Organ 50: 53-69. PubMed: 4371739. 4371739 PMC2481225

[21] Priester WA, Mantel N (1971) (Occurrence of tumors in domestic animals. Data from 12 United States and Canadian colleges of veterinary medicine. J Natl Cancer Inst 47: 1333-1344. PubMed: 5120412. 5120412

[22] Schneider R (1978) General considerations, in: Moulton JE, Tumors in Domestic Animals, University of California Press, Berkley. pp. 1-5.

[23] Priester WA, McKay FW (1980) The occurrence of tumors in domestic animals, Ziegler JL Monograph. Bethesda MD: US Dept of Health and Human Services pp. 1-210. 7254313

[24] Hayes HM, Priester WA Jr, Pendergrass TW (1975) Occurrence of nervous-tissue tumors in cattle, horses, cats and dogs . Int J Cancer 15: 39-47. doi:10.1002/ijc.2910150106. PubMed: 165149. 165149

[25] Luginbuhl H, Fankhauser R, McGrath JT (1968) Spontaneous neoplasms of the nervous system of animals. Prog Neurology Surg 2: 85-164.

[26] Rossmeisl JH Jr., Jones JC, Zimmerman KL, Robertson JL (2013) Survival time following hospital discharge in dogs with palliatively treated primary brain tumors. J Am Vet Med Assoc 242: 193-198. doi:10.2460/javma.242.2.193. PubMed: 23276095. 23276095

[27] Candolfi M, Curtin JF, Nichols WS, Muhammad AG, King GD et al. (2007) Intracranial glioblastoma models in preclinical neuro-oncology: neuropathological characterization and tumor progression. J Neuro Oncol 85: 133-148. doi:10.1007/s11060-007-9400-9. PubMed: 17874037. PMC2384236 17874037

[28] Dickinson PJ, LeCouteur RA, Higgins RJ, Bringas JR, Roberts B et al. (2008) Canine model of convection-enhanced delivery of liposomes containing CPT-11 monitored with real-time magnetic resonance imaging: laboratory investigation. J Neurosurg 108: 989-998. doi:10.3171/JNS/2008/108/5/0989. PubMed: 18447717. 18447717

[29] Dickinson PJ, LeCouteur RA, Higgins RJ, Bringas JR, Larson RF et al. (2010) Canine spontaneous glioma: a translational model system for convection-enhanced delivery. Neuro Oncol 12: 928-940 10.1093/neuonc/noq046 PMC2940703 20488958

[30] Pluhar GE, Grogan PT, Seiler C, Goulart M, Santacruz KS et al. (2010) Retrieved onpublished at whilst December year 1111 from Anti-tumor immune response correlates with neurological symptoms in a dog with spontaneous astrocytoma treated by gene and vaccine therapy . Vaccine 28: 3371-3378. 10.1016/j.vaccine.2010.02.082 PMC2854671 20197146

[31] Louis DN, Ohgaki H, Wiestler OD, Cavenee WK, Burger PC et al. (2007) The 2007 WHO classification of tumours of the central nervous system. Acta Neuropathol 114: 97-109. doi:10.1007/s00401-007-0243-4. PubMed: 17618441. 17618441 PMC1929165

[32] York D, Higgins RJ, LeCouteur RA, Wolfe AN, Grahn R et al. (2012) TP53 mutations in canine brain tumors. Vet Pathol 49: 796-801. doi:10.1177/0300985811424734. PubMed: 22002975. 22002975

[33] Debinski W, Gibo DM (2005). Retrieved onpublished at whilst December year 1111 from Fos-related antigen 1 modulates malignant features of glioma cells . Mol Cancer Res 3: 237-249. 10.1158/1541-7786.MCR-05-0004 15831677

[34] Dickinson PJ, Roberts BN, Higgins RJ, Leutenegger CM, Bollen AW et al. (2006) Expression of receptor tyrosine kinases Retrieved onpublished at whilst December year 1111 from VEGFR-1 (FLT-1), VEGFR-2 (KDR), EGFR-1, PDGFRalpha and c-Met in canine primary brain tumours.; Comp Vet Oncol. 4: 132-140 10.1111/j.1476-5829.2006.00101.x 19754810

[35] Debinski W, Obiri NI, Pastan I, Puri RK (1995) A novel chimeric protein composed of interleukin 13 and Pseudomonas exotoxin is highly cytotoxic to human carcinoma cells expressing receptors for interleukin 13 and interleukin 4. J Biol Chem 270: 16775-16780. doi:10.1074/jbc.270.28.16775. PubMed: 7622490. 7622490

[36] Joshi BH, Plautz GE, Puri RK (2000). Retrieved onpublished at whilst December year 1111 from Interleukin-13 receptor alpha chain: a novel tumor-associated transmembrane protein in primary explants of human malignant gliomas . Cancer Res 60: 1168-1172. 10728667

[37] Balyasnikova IV, Wainwright DA, Solomaha E, Lee G, Han Y et al. (2012) Characterization and immunotherapeutic implications for a novel antibody targeting interleukin (IL)-13 receptor α2. J Biol Chem 287(36): 30215-30227. doi:10.1074/jbc.M112.370015. PubMed: 22778273. 22778273 PMC3436275

[38] Nguyen V, Conyers JM, Zhu D, Gibo DM, Dorsey JF et al. (2011) Retrieved onpublished at whilst December year 1111 from IL-13Rα2-Targeted Therapy Escapees: Biologic and Therapeutic Implications . Transl Oncol 4: 390-400. 10.1593/tlo.11175 PMC3243662 22191003

[39] Brown CE, Starr R, Aguilar B, Shami AF, Martinez C et al. (2012) Stem-like tumor-initiating cells isolated from IL-13Rα2 expressing gliomas are targeted and killed by IL13-zetakine-redirected T Cells. Clin Cancer Res 18: 2199-2209. doi:10.1158/1078-0432.CCR-11-1669. PubMed: 22407828. 22407828 PMC3578382

[40] Binda E, Visioli A, Giani F, Lamorte G, Copetti M et al. (2012) The EphA2 receptor drives self-renewal and tumorigenicity in stem-like tumor-propagating cells from human glioblastomas. Cancer Cell 22(6): 765-780. doi:10.1016/j.ccr.2012.11.005. PubMed: 23238013. 23238013 PMC3922047

[41] Sampson JH, Archer G, Pedain C, Wembacher-Schröder E, Westphal M et al. (2010) Poor drug distribution as a possible explanation for the results of the PRECISE trial. J Neurosurg 113: 301-309. doi:10.3171/2009.11.JNS091052. PubMed: 20020841. 20020841

